# Design of a Guided Internet- and Mobile-Based Intervention for Internet Use Disorder—Study Protocol for a Two-Armed Randomized Controlled Trial

**DOI:** 10.3389/fpsyt.2020.00190

**Published:** 2020-03-17

**Authors:** Karina Saruhanjan, Anna-Carlotta Zarski, Michael Patrick Schaub, David Daniel Ebert

**Affiliations:** ^1^Clinical Psychology and Psychotherapy, Friedrich-Alexander-Universität Erlangen-Nürnberg, Erlangen, Germany; ^2^Swiss Research Institute for Public Health and Addiction, University of Zurich, Zurich, Switzerland; ^3^Department of Clinical, Neuro, and Developmental Psychology, Vrije Universiteit Amsterdam, Amsterdam, Netherlands

**Keywords:** internet- and mobile-based intervention, self-help, internet use disorder, randomized controlled trial, study protocol

## Abstract

**Context:** Internet Use Disorder (IUD), characterized as the inability to control one's internet use, is emerging as an increasing societal concern as it is associated with reduced quality of life and mental health comorbidities. Evidence-based treatment options are, however, scarce due to the novelty of the diagnosis. Internet- and mobile-based interventions may be an effective means to deliver psychological treatment to individuals with IUD as they address individuals affected in their online setting. The aim of the study is to evaluate the efficacy of a newly developed, guided internet- and mobile-based intervention for IUD.

**Methods:** In a two-armed randomized controlled trial (*N* = 130), individuals showing problematic internet use patterns (Internet Addiction Test ≥ 49) will be randomly allocated to the internet- and mobile-based intervention or a waiting control group. Assessments will take place at baseline, 7 weeks, 6- and 12 months after randomization. The primary outcome is internet addiction symptom severity (IAT) at 7 weeks. Secondary outcomes include quality of life, depressive symptoms, anxiety, and other psychosocial variables associated with IUD.

**Intervention:** The intervention consists of seven sessions: Goal setting and motivational interviewing, impulse control, problem solving, cognitive restructuring, self-worth, relapse prevention, and a booster session. Participants are supported by an eCoach who provides individual feedback after completion of each session. Participants can choose between several elective sessions based on individual need.

**Conclusions:** This is the first study to evaluate an internet- and mobile-based intervention for IUD, which could be a promising first step to reduce individuals' disease burden.

**Trial Registration:** DRKS00015314.

The study is currently ongoing. First participants were enrolled in the study on September 14th 2018. Recruitment will continue approximately through March 2020.

## Introduction

Internet Use Disorder (IUD) is characterized by excessive or poorly controlled preoccupations, urges, or behaviors regarding computer use and internet access that lead to social or work-related impairment or distress (1). It includes both excessive gaming and non-gaming internet activities. Non-gaming internet activities can be differentiated in internet gambling, internet pornography, information overload (obsessive research and surfing), internet compulsive buying (2), and excessive social media use. (3, 4). Surveys have indicated that IUD affects 1.5–8.2% of the general population (1, 5). IUD prevalence rates are, however, highly inconsistent due to the varying definitions.

Individuals with IUD show significant social, physical, and mental burdens. IUD may also cause neurological complications, psychological distress, and social problems (6–8). In addition, high comorbidity with psychiatric disorders have been reported, especially affective disorders, anxiety disorders, impulse control disorders, substance use disorders, and attention deficit hyperactivity disorder (1, 9–11). Impairment caused by IUD has also been found to include educational failure, reduced academic perspectives (12, 13), and functional impairment (14).

Despite the severe burden of disease, the range of available specialized evidence-based treatment options for IUD is extremely rare. Treatment accessibility for IUD is impeded as it has only been unsystematically treated so far by selected addiction counseling or educational counseling (15). Currently, there are only very few empirical studies evaluating IUD treatment approaches meeting the scientific standards for randomized controlled trials (16). It has been shown that cognitive-behavioral treatments have large and robust effects on the symptoms of IUD, outperforming other treatments. Among the researched psychological treatments for IUD, none of them measures the effect of an internet-based treatment approach (16). Low utilization of treatment is caused not only by structural but also attitudinal barriers such as fear of stigmatization and low outcome expectancies (17, 18). Internet- and mobile-based interventions can offer a possibility to deliver specialized treatment with a low threshold for uptake.

Treating IUD via the internet may appear contradictive at first. However, there are many arguments supporting this approach: (1) Reaching the target group through their common online setting could be effective as the internet is easily accessible and attractive. (2) Since the target group usually shows low levels of treatment motivation (19), an easily accessible and attractive treatment option is crucial; thus, a low-threshold intervention with interactive audio-visual content may facilitate access and reduce burdens of help-seeking. (3) Due to the individually tailored and adaptable content, a variety of symptoms associated with IUD such as procrastination, bad sleep patterns or alcohol consumption can be addressed. Based on these comorbid symptoms, secondary outcome measures were chosen to assess a reduction in symptoms associated with IUD that were targeted in the intervention. (4) Internet- and mobile-based interventions might be a feasible means to provide evidence-based treatment on a large-scale basis, since they have been shown to be effective in the treatment of numerous mental health disorders (20–23). Therefore, internet- and mobile-based interventions could be a way to reach individuals with IUD better than traditional approach. To the best of our knowledge, this is the first trial evaluating a guided internet- and mobile based intervention for IUD in an RCT design.

## Aims of the Study

The aim of this study is to evaluate the efficacy of a cognitive-behavioral guided self-help internet- and mobile-based intervention for reducing symptoms of IUD compared to a waiting control group (WCG). Both groups have unlimited access to treatment as usual in routine mental health care. It is hypothesized that the intervention reduces symptoms of IUD.

## Methods

### Design

**Figure 1:**
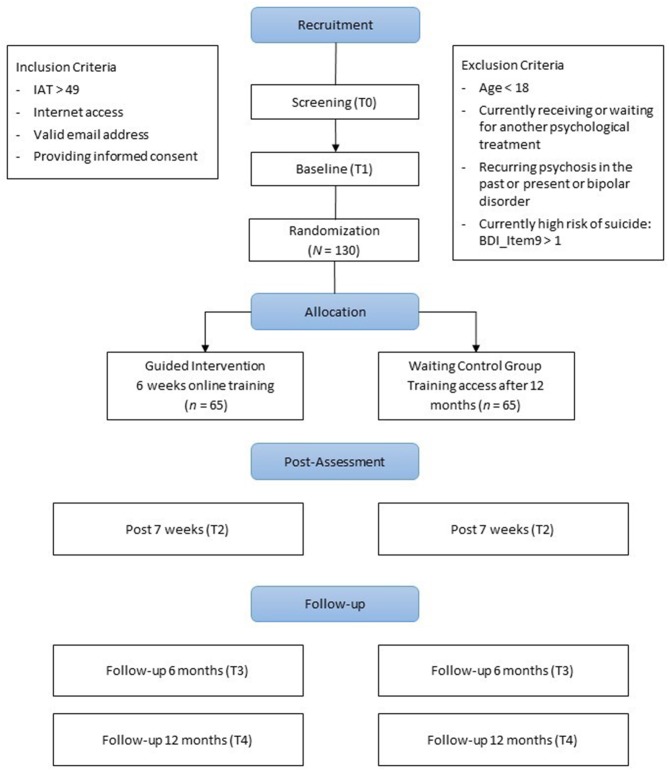
Study flow chart.

A two-arm randomized controlled trial (RCT) will be conducted to evaluate the internet- and mobile-based intervention compared to a WCG. Assessments will take place at baseline (t1), post-intervention (t2), at 6- (t3) and 12-months follow-up (t4). See Figure 1 for a detailed overview of the study design. All procedures involved in the study will be consistent with the generally accepted standards of ethical practice approved by the Friedrich-Alexander University of Erlangen-Nuremberg ethics committee (54_18 B).

### Participants and Procedures

#### Inclusion and Exclusion Criteria

We will include individuals who (1) are at minimum 18 years of age, (2) show elevated levels of IUD with scores of ≥ 49 on the Internet Addiction Test (IAT) (7), (3) have internet access, (4) have sufficient German language reading and writing skills and (5) are willing to give informed consent. We will exclude subjects who (1) indicate that they have been diagnosed with a psychosis or bipolar disorder, (2) show a notable suicidal risk as indicated by a score greater than 1 on Item 9 of the Beck Depression Inventory (BDI) (24), (3) currently receive or are on a waitlist for psychological treatment regarding any mental disorder.

#### Recruitment

Participants will be recruited in Germany, Austria and Switzerland via (1) the GET.ON Website, (2) the StudiCare website, (3) recruitment over social media and discussions forums, and (4) mass e-mailing with information regarding the study and intervention sent to German, Swiss and Austrian psychological counseling centers and university students (in Erlangen, Ulm, Bern, Dresden, Hagen and Vienna).

#### Assessment of Eligibility and Randomization

After registering with a self-chosen email address, applicants will receive an email with detailed information about the study procedure. They will be further informed that it is possible to withdraw from the intervention and/or study at any time without any negative consequences. Applicants are asked to complete an online screening questionnaire and to sign the informed consent form and return it by post or email. As soon as participants have completed the baseline assessment and meet the inclusion criteria, they will enter the study and will be randomized to one of the two study conditions. Participants are randomized to either the guided intervention group or a WCG in a 1:1 ratio. Randomization is performed by a research assistant not otherwise involved in the study using block randomization with varying block sizes. Once randomization has been completed, participants in the intervention group receive immediate access while participants in the control group receive access 12 months later.

### Intervention

The intervention consists of seven sessions: Goal setting and motivational interviewing (session 1), impulse control (session 2), problem solving (session 3), cognitive restructuring (session 4), self-worth (session 5), relapse prevention (session 6), and a booster session 4 weeks after completion of the core sessions (session 7) (see Table 1 for an overview of the main sessions). Moreover, participants can choose between several elective sessions based on individual need and preference. The elective sessions are directed at personal needs and values, sleep, relaxation, alcohol and affect regulation, appreciation and gratefulness, and procrastination (see Table 2 for an overview of the elective sessions). Each session can be completed in ~30–45 min. We advise participants to do at least one and a maximum of two sessions per week. Further content can only be accessed once previous sessions are completed. Consequently, the training will last about 4–6 weeks plus one additional booster session 4 weeks after completion of the last session. The booster session aims to help participants to refresh and reflect contents and strategies. Sessions consist of texts, testimonials, and include many interactive elements such as quizzes, exercises, and homework, which can be seen in the Supplementary Material. At the end of the second session, a plan for behavior change is introduced. This four-step plan aims to help participants to (1) choose specific situations in which they want to change their internet use, (2) set realistic goals, (3) collect ideas on how to realize their goals, and (4) to create a detailed plan for goal acquisition including newly acquired strategies and planning for potential upcoming difficulties. Participants are expected to test their plan for behavior change during the following week and are encouraged to analyze success and difficulties in the training process in the next session. They can also opt to review and adapt their plan in the following session. Participants are encouraged to formulate five plans in total over the course of the training between the second and the sixth session. They are also invited to keep an online diary, which can be accessed via the web interface or an optional smartphone app. The intervention uses content tailoring, thereby engaging the participants by encouraging them to make real-time choices among various response options, and then providing individualized content based on specific needs or preferences. The training is built on responsive web-design and can be completed on any kind on internet-ready device, such as PCs/laptops, smartphones, or tablets. Participants can also opt to receive motivational messages and small exercises referred to as *Tiny Tasks*. These smartphone notifications are sent directly to their mobile device 3 times a day. These notifications will support the participants in transferring the exercises of the training into their daily lives (e.g., giving suggestions on how to implement strategies: “What has influenced your internet usage today?”).

**Table 1:** Content of the training.

**Intervention content**	**Session**
Goal setting and motivational interviewing	1
Impulse control	2
Problem solving	3
Cognitive restructuring	4
Self-worth	5
Relapse prevention	6
Booster session	7

**Table 2:** Content of the elective sessions.

**Session**	**Content**
Relaxation	Learning the progressive muscle relaxation to reduce tension
Alcohol & affect regulation	Reducing affect regulation related alcohol consumption
Personal needs & values	Reducing personal incongruence in daily life, by achieving balance between personal needs and values
Appreciation & gratefulness	Focusing on the good things and practicing mindfulness strategies
Sleep	Improving sleep hygiene and practicing sleep restriction to enhance sleep quality
Procrastination	Practicing strategies addressing delaying important tasks

#### Goal Setting and Motivational Interviewing (Session 1)

The first session gives an overview of internet usage in everyday life (e.g., social media, news, online dating), the upcoming sessions, and basic techniques of the intervention. To support participants, three fictional patients are introduced who vary in age, gender and ethnicity, being addicted to online gaming, social media or online dating. One of the main elements of the first session is psychoeducation on the subject of IUD, including the difference between common and problematic internet use, and epidemiology of the disease. Based on that, elements of the motivational interviewing are implemented such as elaborating on personal advantages and disadvantages of using the internet, individual motivation for and confidence in behavior change. Finally, participants are encouraged to create a precise treatment goal using the SMART model.

#### Impulse Control (Session 2)

The second session introduces the concept of impulse control and self-control in order to help the participants to handle feelings of craving to go online in everyday life. Participants can choose between different exercises, each of them illustrating one strategy [(1) distraction, (2) positive self-instruction, (3) social support, (4) awareness of consequences, (5) stimulus control, (6) mindfulness, and consciousness of the presence]. Afterwards, participants are invited to learn about the benefit of their individual resources (e.g., physically, psychologically, socially) regarding coping of daily hazzles or crisis and implementation of strategies. Finally, they are presented with a list of positive activities offering alternatives to the internet and can create a detailed plan when and where to implement the activities.

#### Problem Solving (Session 3)

In the third session, participants are presented with strategies on how to deal with difficulties to stay offline. These strategies address motivational problems, fear of missing out on social situations online, problems in accepting oneself in real life, and loosing social integration online. To enhance their understanding of their problems, participants are introduced to the TRIAS disorder model (25). Based on the TRIAS-Modell participants can then identify individual influencing factors on their internet usage such as their environment (e.g., family, challenging conflict situations), personal dispositions and characteristics (e.g., personality, coping strategies), opportunities of the internet (e.g., self-representation, social contacts) and develop specific strategies such as positive self-instructions with the help of their impulse control and self-control techniques. Participants then learn about psychological, physical, and social maintaining factors of the disorder in combination with specific coping techniques such as strengthening their self and their social relationships offline.

#### Cognitive Restructuring (Session 4)

In the fourth session, participants are introduced to cognitive restructuring, receiving information on the causal relationship between cognitions, emotions, behavior, and consequences. Participants can then individualize their personal thought records by including personal situations, cognitions, and emotions. After psychoeducation about negative emotions and acceptance, participants create positive and helpful thoughts to contrast their negative thoughts in their thought record. To generally concentrate on and practice positive thinking habits, strategies as taking notes or using cues to remind themselves of helpful thoughts and imagining, how helpful thought would alter the current situation.

#### Self-Worth (Session 5)

This session aims at highlighting the importance of self-confidence, self-worth and self-efficacy concerning meeting challenges and controlling internet usage. First, participants learn how their internet use can affect their self-worth and vice versa. To strengthen their self-worth, participants are initially invited to state their positive characteristics and skills. After that, the concept of the inner critic, representing self-doubting cognitions, and the benevolent companion, representing self-conductive cognitions, are introduced and participants are encouraged to identify their own self-doubting and negative self-deprecating cognitions, and confront them with their benevolent companion. Lastly, participants plan the upcoming days with activities that foster self-care, such as spending time with friends and family or pursue hobbies.

#### Relapse Prevention (Session 6)

In the last session, participants can review brief summaries of each session. They are asked to reflect on their progress concerning their intervention goals, and to identify mechanisms of their individual behavior change during the intervention as well as helpful psychological strategies. Subsequently, they are encouraged to make a specific plan of strategies they want to continue exercise in everyday life until the booster session, in order to maintain and generalize acquired strategies.

#### Booster Session (Session 7)

The participants are invited to complete a booster session 4 weeks after completion of the sixth session. In this session, they can reflect on their learning experience and personal goal attainment. They are asked to consider their current use of the internet, and are provided with additional information on support, if needed. Participants can review the letter they wrote to themselves and set new goals for the upcoming months.

#### Guidance

During the active intervention phase, participants of the treatment arm receive content-focused guidance (26) by an eCoach who provides individual manualized feedback after completion of each session. eCoaches will have at least a Bachelor's degree in Psychology and have access to supervision when required. Guidance is based on a treatment manual with preformulated standardized text blocks that are prepared for every lesson and individually adapted for participants according to their input and overall progress. As an additional feature, participants have the chance to contact their eCoach through the internal messaging function of the platform. Estimated mean time per feedback is 30 min. eCoaches are advised not to use more than 40 min per individual feedback. Approximately 2.5 h is the total time an eCoach will spend per participant. Session adherence is also monitored, when participants do not complete a session within 7 days, eCoaches will send out email reminders after 7, 14, and 21 days. Additionally, after 28 days a reminder will be sent via text message. Reminders have shown to improve adherence to self-guided behavior change interventions (27, 28). All study participants can contact a support email address in case of any technical difficulties regarding the intervention.

### Assessment and Data Management

Self-reports will take place at screening (T0), baseline (T1) prior to randomization, post-intervention (T2) 7 weeks after randomization, at 6- (T3) and 12-month follow-ups (T4; see Figure 1 for a detailed overview). Self-reported data will be collected using a secure online-based assessment system (AES, 256-bit encrypted).

### Outcomes

#### Primary Outcome

In order to assess the effect of the treatment on symptoms of IUD, the Internet Addiction Test [IAT; (7)] is administered. The IAT is a widely accepted and validated testing instrument that examines a variety of symptoms of internet dependency (29). The 20 items assess, on a 5-point Likert Scale (1 = “rarely,” 2 = “occasionally,” 3 = “frequently,” 4 = “often,” 5 = “always”) the participant's preoccupation with the internet (e.g., “How often do you block out disturbing thoughts about your life with soothing thoughts of the internet?”), excessive use (e.g., “How often do you lose sleep due to late-night logins?”), neglection of work and social life (e.g., “How often does your job performance or productivity suffer because of the internet?”, “How often do you form new relationships with fellow on-line users?”), anticipation (e.g., “How often do you find yourself anticipating when you will go online again?”), and loss of control (e.g., “How often do you try to cut down the amount of time you spend on-line and fail?”). In several studies, the scale has been shown very good internal consistencies (α = 0.91, α = 0.89) (30).

#### Secondary Outcomes

##### Compulsive internet use

The Compulsive Internet Use Scale (CIUS) (31) consists of 14 items, which refer particularly to the compulsive and impulse control elements of internet use, assessing the severity of symptoms on a 5-point Likert Scale. In several studies, the CIUS has proven to be a valid instrument and has shown good internal consistency (α = 0.89) (32). The scales include withdrawal symptoms, loss of control, preoccupation/salience, conflict (referring to social and work life) and coping (using the internet to cope with stressors).

##### Depressive symptoms

Self-reported depressive symptoms will be measured with the Patient Health Questionnaire (PHQ-9) (33). This frequently used self-report instrument consists of 9 items that are answered on a 4-point Likert scale referring to the previous 2 weeks. Total scores range from 0 to 27. The internal consistency of this measure has been found to be excellent (α = 0.83–0.92) (34).

##### Anxiety

The generalized anxiety disorder measurement (GAD-7) is used to assess symptom severity (35). It consists of 7 items answered from 0 (not at all) to 3 (nearly every day). It possesses excellent internal consistency (α = 0.92) and good test-retest reliability (Intraclass Correlation Coefficient = 0.83) (35, 36).

##### Problematic alcohol consumption

We will use the 3-item version of the Alcohol Use Disorder Identification Test (AUDIT-C) to measure problematic alcohol consumption of the participants on a 5-point Likert Scale (37). The AUDIT-C is a validated and widely used brief screening test for heavy drinking, alcohol abuse, or dependency (38). Even though the internal consistency is only satisfactory (α = 0.77–0.80), it is outweighed by the brevity of the AUDIT-C (39).

##### Insomnia

Insomnia severity will be assessed by the Insomnia Severity Index (ISI) (40), consisting of 7 items to be answered on a 5-point Likert scale. Psychometric properties have been shown to be good (α = 0.83) (41).

##### Worries

In order to evaluate worries, the ultra-brief version of the Penn State Worry Questionnaire (PSWQ-3) (42) is applied. It is a questionnaire that assesses self-reported key aspects of worry, consisting of three items, which are rated on a 5-point Likert Scale. Cronbach's alpha has shown to be 0.74 (43).

##### Procrastination

The 9-item-version of the General Procrastination Scale (GSP-K) is administered to measure procrastination behavior on a 4-point Likert Scale (44, 45). Overall, the questionnaire shows a very good internal consistency (α = 0.92).

##### Gambling

The German questionnaire Kurzfragebogen zum Glücksspielverhalten (KFG) (46) addresses lifetime gambling behavior and consists of 20 items, each on a 4-point Likert Scale. The threshold for pathological gambling is set at 16 points. The questionnaire shows a satisfactory internal consistency (α = 0.79) (47).

##### Well-being

Well-being will be assessed by the 5-item WHO-5 Well-Being Index (WHO-5) (48) answered on a 6-point Likert Scale with scores ranging from 0 to 30 (α = 0.82) (49).

##### Quality of life

To measure quality of life, we will use the Assessment of Quality of Life Instrument (AQoL-8D) (50, 51), which consists of 35 items with eight dimensions: independent living (α = 0.9, ICC = 0.86), pain (α = 0.85, ICC = 0.86), senses (α = 0.69, ICC = 0.51), mental health (α = 0.84, ICC = 0.89), happiness (α = 0.85, ICC = 0.90), coping (α = 0.80, ICC = 0.79), relationships (α = 0.73, ICC = 0.88), self-worth (α = 0.85, ICC = 0.81) (51). Psychometrics properties have been shown to be acceptable.

##### Work limitations

To measure the on-the-job impact of chronic health problems and/or treatment with a focus on assessing limitations while performing specific job demands, the Work Limitations Questionnaire (WLQ) (52) is applied. It consists of 25 items on a 5-point Likert Scale, assessing four dimensions of work limitation. Walker et al. (53) report a Cronbach's alpha range of 0.83–0.88 (53).

##### Costs associated with psychiatric illness

Other measures related to the intervention include the Trimbos Questionnaire for costs associated with psychiatric illness (TiC-P) (54), including healthcare utilizations and productivity losses, adapted to the German health care system. It consists of 14 yes/no questions to assess contacts with healthcare providers of any kind and five items covering reduced efficiency and/or absence from work due to a psychiatric condition. The TiC-P has been proven to be a feasible and reliable instrument for measuring healthcare utilization and productivity loss (55). The German adaption has been utilized in a substantial number of randomized controlled trials as a basis for health economic outcome evaluations (56–67).

##### Training and acceptability

User satisfaction will be assessed by a questionnaire based on the Client Satisfaction Questionnaire (CSQ-8) (68), adapted to assess user satisfaction in online interventions (69). Global client satisfaction with the internet- and mobile-based intervention is measured by eight items. Previous research indicated good psychometric properties (α = 0.84–0.97) (70).

#### Other Assessments

Other assessments include demographics (e.g., age, gender, occupation, level of education) and current and previous experience with psychotherapy, self-esteem via Rosenberg Self-Esteem Scale (RSES) (71) and social phobia via Mini Social Phobia Inventory (Mini-SPIN) (72). For an overview of all outcome measures, see Table 3.

**Table 3:** Overview study self-report assessments.

**Construct**	**Questionnaire**	**T1**	**T2**	**T3**	**T4**
Demographics	Socio-Demographic Data	✓	–	–	–
Internet addiction	Internet Addiction Test (IAT)	✓	✓	✓	✓
	Compulsive Internet Use Scale (CIUS)	✓	✓	✓	✓
Depression	Patient Health Questionnaire (PHQ-9)	✓	✓	✓	✓
Anxiety	General Anxiety Disorder Measurement (GAD-7)	✓	✓	✓	✓
Alcohol abuse	The Alcohol Use Disorders Identification Test (AUDIT-C)	✓	✓	✓	✓
Sleep	Insomnia Severity Index (ISI)	✓	✓	✓	✓
Worries	Penn State Worry Questionnaire – Ultra Brief Version (PSWQ-3)	✓	✓	✓	✓
Self-esteem	Rosenberg Self Esteem Scale (RSES)	✓	–	–	–
Social phobia	Social Phobia Inventory (Mini-SPIN)	✓	–	–	–
Procrastination	General Procrastination Scale—Short Version (GPS-K)	✓	✓	✓	✓
Gambling	Kurzfragebogen zum Glücksspielverhalten (KFG)	✓	✓	✓	✓
Wellbeing	WHO-5 Wellbeing Index (WHO-5)	✓	✓	✓	✓
Quality of life	Assessment Quality of Life (AQoL-8D)	✓	✓	✓	✓
Work limitations	Work Limitations Questionnaire (WLQ)	✓	✓	✓	✓
Health economic evaluation	Trimbos Questionnaire for Costs Associated with Psychiatric Illness (TiC-P)	✓	–	✓	✓
Training and acceptability	Client Satisfaction Questionnaire (CSQ-8)	–	✓	–	–

*T1, baseline; T2, post-intervention; T3, 6-month follow-up; T4, 12-month follow-up*.

#### Response

To determine the numbers of participants achieving a reliable positive outcome, we will code participants as responders or non-responders according to the widely used reliable change index (RCI) (73) after participation in the intervention. Participants will be considered responders when they display an RCI score of above 1.96.

### Statistical Methods

Data will be analyzed on an intention-to-treat basis including all participants who will be randomly assigned to conditions. Additionally, study completer analyses including only participants who filled out the questionnaires and intervention completer analyses including only participants who have completed at least four out of six sessions will be conducted. Missing data will be handled using multiple imputations (MIs) calculated by a Markov Chain Monte Carlo multivariate imputation algorithm of 100 estimations per missing value. MI is especially robust with respect to missing data. We will conduct an ANCOVA to examine differences in the primary outcome. To detect differences between the two study groups, we will conduct univariate analysis of covariance to compare secondary outcomes at post-treatment and 6- and 12-month follow-up adjusting for baseline scores. Predictors and mediators of changes of the primary outcome will be analyzed on exploratory basis using linear regression. For all analyses on continuous measures, Cohen's d (74) will be calculated by standardizing the differences between baseline and follow-up scores by the pooled standard deviation of the baselines scores.

The response rates will be compared across conditions with the help of contingency tables and Chi-Squared tests. For primary analysis, significance levels will be set at 0.05, one-sided. For the explorative analysis of the data, the significance levels will be set to 5%. All analyses will be performed with IBM SPSS v. 24.

### Sample Size Calculation

To answer the research question described above, we aim to include 130 participants. That is to statistically detect a medium effect of (Cohen's d) *d* = 0.60, with a power (1–β) of 80% and an α of 0.05 (two-tailed test) for an intention-to-treat analysis using G^*^Power (75). The estimated effect of (Cohen's d) *d* = 0.60 is based on recent meta-analyses on the effects of treatments on internet addiction which shows rather high effect sizes for CBT (16) as well as several other psychological treatments (76).

## Discussion

The aim of this two-armed randomized controlled trial is to evaluate the efficacy of a newly developed internet- and mobile-based intervention for IUD in comparison to a WCG. It is hypothesized that the intervention decreases symptoms of IUD in the intervention group as compared to the WCG.

IUD is associated with a high disease burden, a decline in quality of life, and substantial comorbidities. Yet, treatment options are very scarce and unspecialized (15). This study provides some of the first guidelines for the development and efficacy of internet-based treatment for IUD. The intervention is designed as a low-threshold approach and adapted to various internet activities as part of IUD, comorbidities and cultural backgrounds through testimonials. Though treating IUD with the internet appears contradictive at first, it bears great potential because affected individuals are addressed in their online setting. An internet- and mobile-based intervention is easily accessible, anonymous, therefore likely to lower the threshold of treatment utilization and thus can be attractive to the target group which is usually characterized by low treatment motivation. Furthermore, to create an engaging and motivating intervention, adaptable content and multiple elective components of motivating and educative nature are used. The intervention structure is set up easy to follow and individual tailored. Content tailoring allows participants to make real-time choices, which trigger different content based on preference or need. To account for the quality of the intervention, participants are provided guidance by an eCoach. Besides the supportive and engaging function of the eCoach within the treatment process, guidance also has been shown to increase adherence rates in internet-and mobile-based interventions (26). To increase treatment adherence, eCoaches send participants feedback on completed sessions and are available for consultation. Moreover, we seek to foster the transfer of strategies into daily life, e.g., by sending tiny tasks via app. A strength of our treatment approach is that the intervention aims at reducing internet use and foster a controlled internet usage instead of establishing abstinence. Participants are invited to define their treatment goals on their own and decide about the way they want to change their internet use and how much time they allow themselves to use the internet. Thus, we aim to set up and motivate individuals into a therapeutic process involving participants and fostering self-determination. Further strengths of the study include the strong methodology of a randomized controlled design compromising an appropriate statistical analyses plan with missing data handling with state of the arts methods (77). Thus, this study will contribute significantly to the literature and empirically tested treatment for IUD because, to the best of our knowledge, it is the first study to investigate the efficacy of a guided internet- and mobile-based intervention for the treatment of IUD.

This study also has some limitations. The power analyses was calculated with an estimated effect of *d* = 0.60. It should be taken into account that smaller effects can be clinically relevant as well. We will not include any additional objective measurement of IUD, e.g., tracking of time spent online. To allow a low-threshold approach, only self-reported measurements are used. Future research should consider additional objective measures and independent ratings of internet use and of symptoms associated with IUD. As motivation for treatment is typically low in the target group and guided internet- and mobile-based interventions require high levels of self-regulation, we account for drop-out with a systematic adherence protocol including reminders via email after 7, 14, and 21 days in case participants do not complete a measurement point followed by telephone calls. Moreover, we provide monetary incentives for completing the online questionnaires. With regard to the pitfall of a usually overeducated sample of participants in internet- and mobile-based interventions, we aim to recruit at a broad level in the population, including health insurance companies, counseling centers and local community centers.

## Conclusion

To conclude, this internet- and mobile-based intervention is a treatment that aims to reduce disease burden of IUD in the general population. To complement research on IUD and to advance evidence-based treatment options, RCTs evaluating treatments for IUD are needed. Internet- and mobile-based interventions might be an appropriate strategy to overcome low treatment availability. This study will contribute to empirical research on IUD and provide information about their acceptability and efficacy. If the efficacy of this intervention can be established, the effectiveness of the intervention needs to be evaluated with regard to large-scale implementation in routine care to make the intervention available to a large number of affected individuals irrespective of place and time.

## Ethics Statement

This study has been approved by the ethics committee of the Friedrich-Alexander University Erlangen Nürnberg (no. 54_18 B). Written informed consent will be obtained from the participants of our study.

## Author Contributions

KS and DE designed the study. KS drafted the manuscript supervised by A-CZ. DE and MS contributed to the further writing of the manuscript. All authors read and agreed to be accountable for all aspects of the work ensuring that questions related to the accuracy or integrity of any part of the work are appropriately investigated and resolved.

### Conflict of Interest

DE reports to have received consultancy fees or served in the scientific advisory board of Minddistrict, Sanofi, Novartis, Lantern, Schön Kliniken, and German health insurance companies (BARMER, Techniker Krankenkasse). He is also stakeholder of the Institute for health trainings online (GET.ON), which aims to implement scientific findings related to digital health interventions into routine care. The remaining authors declare that the research was conducted in the absence of any commercial or financial relationships that could be construed as a potential conflict of interest.
