# Supplementary material for: Design of a Guided Internet- and Mobile-Based Intervention for Internet Use Disorder—Study Protocol for a Two-Armed Randomized Controlled Trial
Source: Front Psychiatry. 2020 Mar 17;11:190. doi: 10.3389/fpsyt.2020.00190 (PMC7092751; doi:10.3389/fpsyt.2020.00190)
Supplement: Supplementary file 1 [file Data_Sheet_1.PDF]

Appendix A

Extract from the intervention Get.On Offline with interactive elements.

Meine Internetnutzung der letzten Woche

Wie viele Stunden hast du in den letzten 7 Tagen durchschnittlich pro Tag im Internet verbracht? (Hierzu zählt nicht die Zeit, in der du das Internet beruflich oder im Rahmen eines Studiums verwendet hast.)

0

0 24

Wie hat es deiner Meinung nach in der letzten Woche bei dir geklappt, deine Internetnutzung zu kontrollieren? (1 = gar nicht, 10 = sehr gut)

1

1 10

Wie zufrieden bist du mit deinem Trainingserfolg in der letzten Woche? (1 = gar nicht, 10 = sehr zufrieden)

1

1 10

Bewerte bitte die Umsetzung dieses Ziels:

- Es lief super!
- Es lief einigermaßen okay.
- Es lief nicht so gut.
- Es hat gar nicht geklappt.

Es ist völlig normal, dass es auch mal nicht so gut läuft, lass dich davon nicht unterkriegen. Du kannst stolz auf dich sein, dass du heute trotzdem wieder dabei bist. Schwierigkeiten gehören auf dem Weg der Veränderung leider auch dazu, sie können anstrengend und kräftezehrend sein, aber geben uns auch die Möglichkeit, aus ihnen zu lernen. Berichte uns doch mal von einer Situation in der letzten Woche, in der du Schwierigkeiten hattest.

Situation	Welche Gedanken hattest du?	Wie hast du dich dabei gefühlt?	Wie stark war dein Verlangen, online zu sein? (0-100%)	Wie lange warst du online? (in Minuten)

+Hinzufügen
